# Supplementary material for: Stimuli-responsive hydroxyapatite liquid crystal with macroscopically controllable ordering and magneto-optical functions
Source: Nat Commun. 2018 Feb 8;9:568. doi: 10.1038/s41467-018-02932-7 (PMC5805687; doi:10.1038/s41467-018-02932-7)
Supplement: Supplementary file 1 — Supplementary Information [file 41467_2018_2932_MOESM1_ESM.pdf]

Supplementary Information

Stimuli-responsive hydroxyapatite liquid crystal with macroscopically controllable ordering and magneto-optical functions

Nakayama et al.

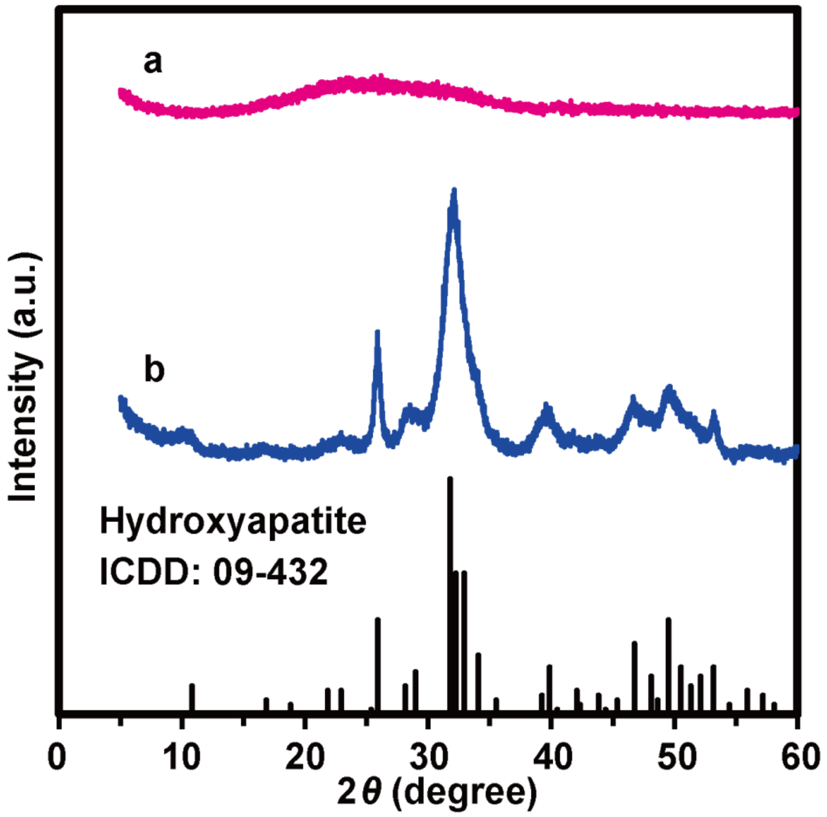

Supplementary Figure 1 X-ray diffraction (XRD) patterns of precipitate in early stage and final product. **a, b** XRD patterns of precipitates collected after (**a**) 10 min and (**b**) 3 days.

Supplementary Note 1

The precipitate formed within 10 min showed no diffraction peaks in the XRD pattern (Supplementary Fig. 1a), suggesting that amorphous calcium phosphate was formed in the early stage. For the precipitate collected after 3 days, diffraction peaks characteristic of hydroxyapatite (HAp) were observed (Supplementary Fig. 1b). These results indicate the formation of HAp crystals through amorphous calcium phosphate precursors.

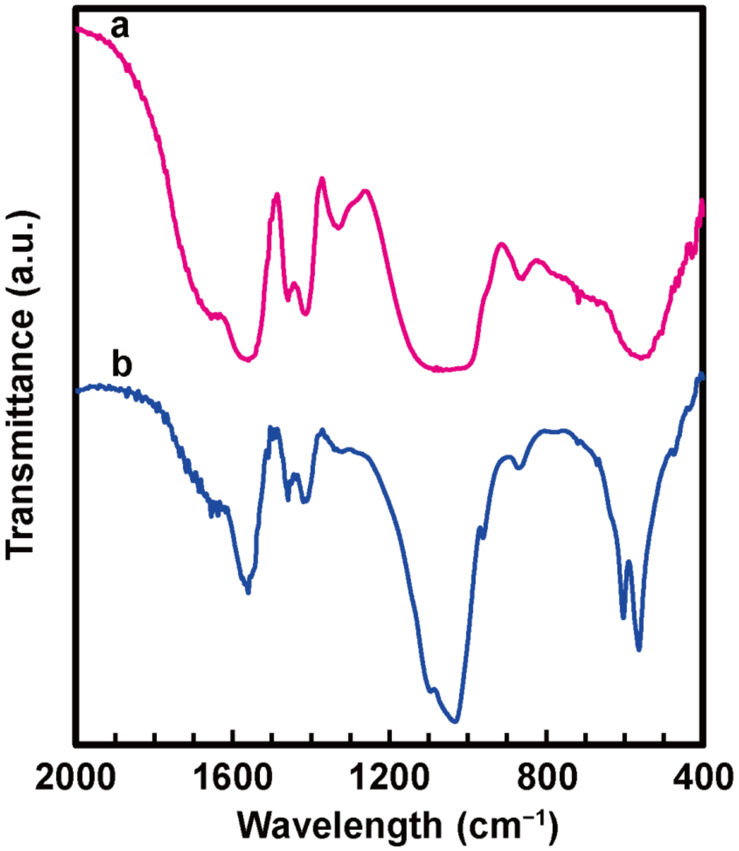

Supplementary Figure 2 Fourier-transform infrared (FTIR) spectra of precipitate in early stage and final product. **a, b** FTIR spectra of precipitates collected after (**a**)10 min and (**b**) 3 days.

Supplementary Note 2

The precipitates obtained after 10 min and 3 days were also identified using FTIR spectroscopy. The broad peaks at 557 cm^{-1} and 1065 cm^{-1} (Supplementary Fig. 2a) are characteristic of amorphous calcium phosphate. After 3 days, the broad peak at 557 cm^{-1} split to give two sharp peaks, at 563 cm^{-1} and 604 cm^{-1} (Supplementary Fig. 2b), which are ascribed to the ν_3 vibration and ν_4 bending mode of HAp[1]. These results are consistent with the XRD patterns (Supplementary Fig. 1). The peaks in the range 1400–1600 cm^{-1} are attributable to the absorption of the carboxylate groups of poly(acrylic acid) (PAA).

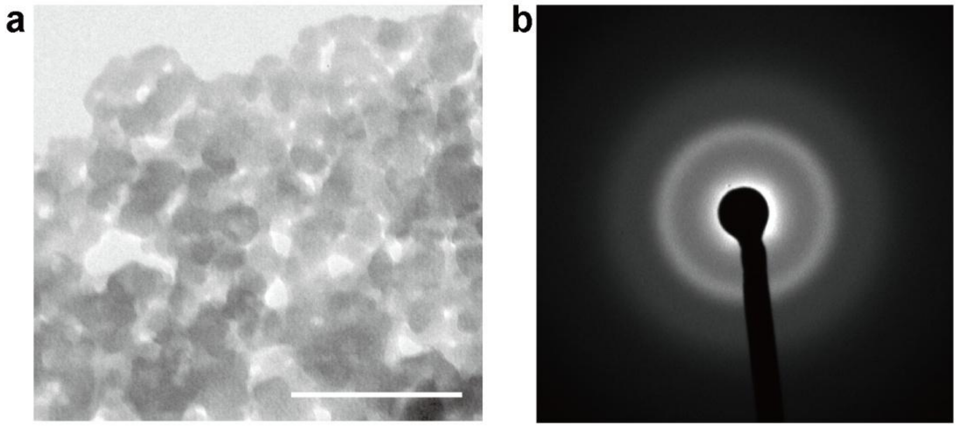

Supplementary Figure 3 Transmission electron microscopic (TEM) images of precipitate in early stage. **a** TEM image of precipitates collected after 10 min and **b** corresponding selected-area electron diffraction (SAED) pattern. Scale bar, 100 nm.

Supplementary Note 3

As shown in Supplementary Fig. 3a, the calcium phosphate precursors were nanoparticles with diameters around 10–30 nm. The SAED pattern (Supplementary Fig. 3b) shows broad ring patterns, suggesting that the nanoparticles had amorphous-like structure.

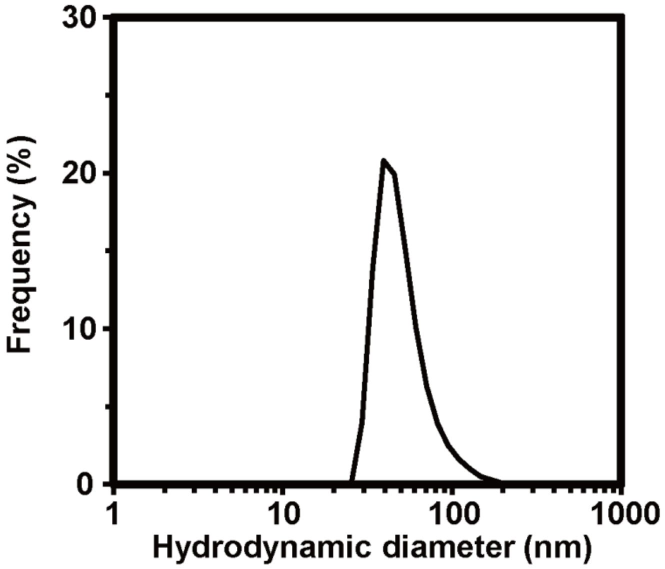

Supplementary Figure 4 Dynamic light scattering (DLS) measurements for amorphous precursors. DLS measurements for aqueous colloidal dispersions of amorphous calcium phosphate precursors.

Supplementary Note 4

DLS measurements (Supplementary Fig. 4) showed that the average diameter of the amorphous calcium phosphate nanoparticles was 50 ± 20 nm, which is larger than the diameter observed using TEM. This size discrepancy implies hydration of the nanoparticle precursors in aqueous solution.

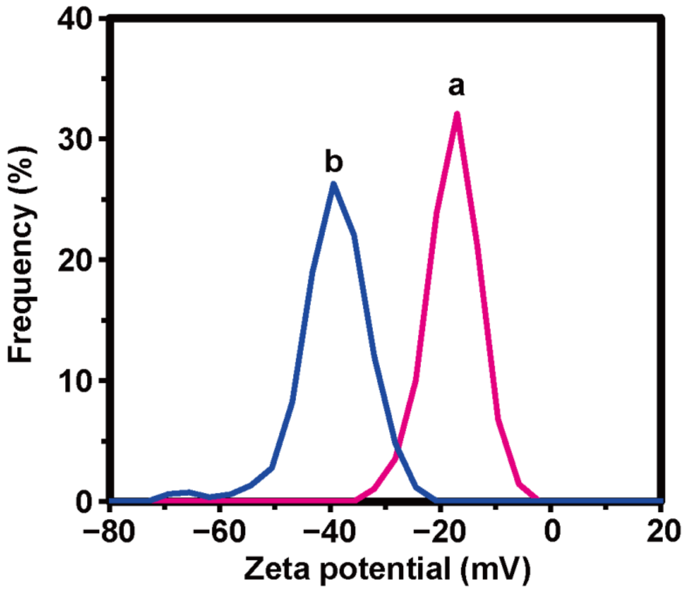

Supplementary Figure 5 Zeta potential measurements for amorphous precursors and HAp nanorods. **a, b** Zeta potential measurements for aqueous colloidal dispersions of (**a**) amorphous calcium phosphate precursors and (**b**) HAp nanorods.

Supplementary Note 5

Zeta potential measurements showed that the surface potentials of the amorphous calcium phosphate precursors was −18 ± 1 mV (Supplementary Fig. 5a). The HAp nanorods had surface potentials of −39 ± 7 mV (Supplementary Fig. 5b). These results indicate that the precursors and nanorods were negatively charged.

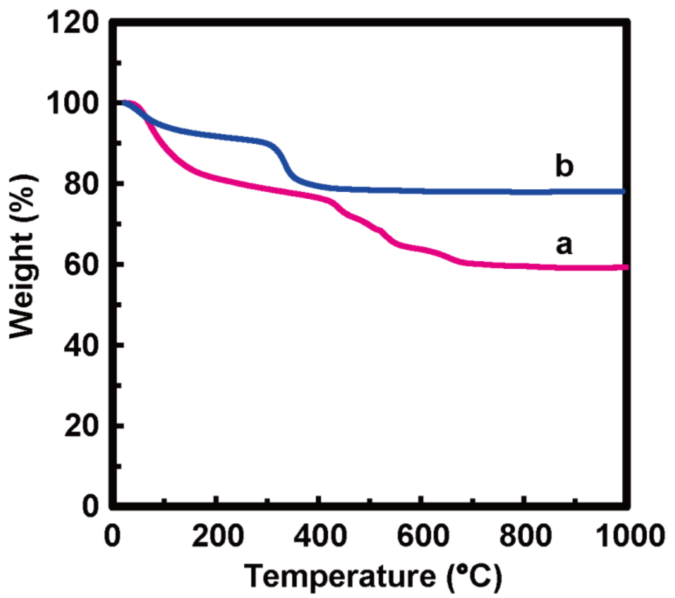

Supplementary Figure 6 a, b Composition analyses for (**a**) amorphous precursors and (**b**) HAp nanorods. Thermogravimetric (TG) measurements were performed on amorphous calcium phosphate precursors and HAp nanorods under air flow (100 mL min^{-1}) after drying them under ambient conditions.

Supplementary Note 6

In the literature[2], it was reported that PAA molecules decompose at 200 °C to 600 °C, and water molecules evaporate below 200 °C. Accordingly, the composition of the amorphous calcium phosphate precursors was calculated to be 63.8 wt% amorphous calcium phosphate, 17.6 wt% PAA, and 18.6 wt% water (Supplementary Fig. 6a). The HAp nanorods consisted of 78.2 wt% HAp, 13.7 wt% PAA, and 8.10 wt% water (Supplementary Fig. 6b). The surface negative charges of these precursors and nanorods (Supplementary Fig. 5) are ascribed to the presence of PAA molecules.

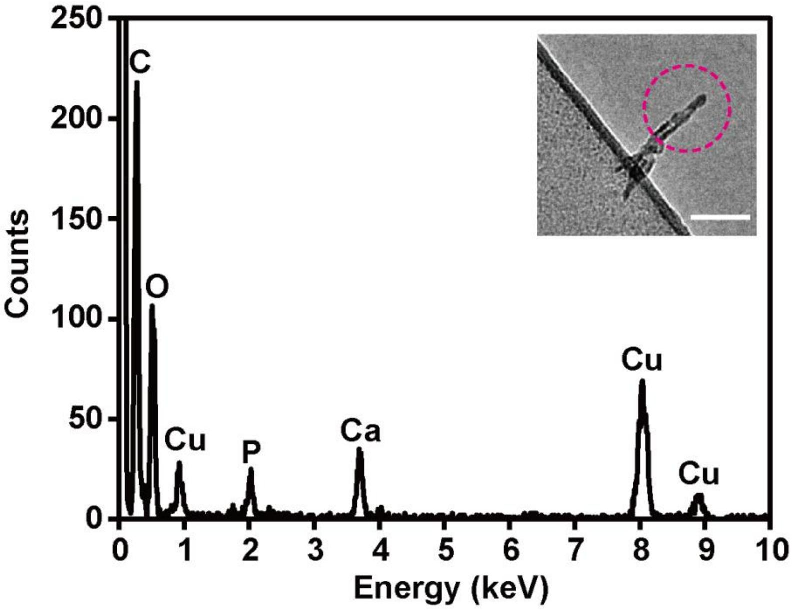

Supplementary Figure 7 Energy-dispersive X-ray spectroscopy (EDS) analysis of HAp nanorods. Elements included in a HAp nanorod were analyzed by EDS analysis. The inset shows a TEM image of a nanorod and the magenta circle indicates the area used for the EDS analysis. Scale bar, 50 nm.

Supplementary Note 7

The EDS analysis shows that Ca, P, C and O elements were included in the HAp nanorod, which are all ascribed to HAp, PAA or water molecules (Supplementary Fig. 7). No impurities were detected in the nanorod. The Cu elements originate from the TEM grid.

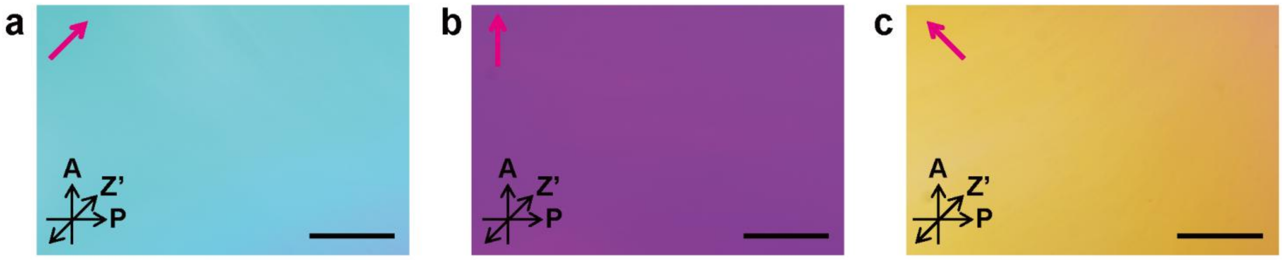

Supplementary Figure 8 Interference color observations for assembly oriented by mechanical shearing. **a–c** Polarizing optical microscopy (POM) images taken with a wave plate on every rotation of the oriented sample by 45°, after mechanical shearing of a liquid-crystalline (LC) colloidal dispersion (9.9 vol%). The magenta arrow in each image indicates the shearing direction. A: Analyzer; P: Polarizer; Z'; slow axis of the wave plate. Scale bars, 1 mm.

Supplementary Note 8

The birefringence Δn of an oriented material is defined as

$$\Delta n = n_{||} - n_{\perp} \quad (1)$$

where $\Delta n_{||}$ and $\Delta n_{\perp}$ are the refractive indices of light that is polarized in a parallel direction and a perpendicular direction, respectively, to the orientation direction[3].

The phase difference between these polarized lights, i.e., the retardation R, is described as

$$R = d\Delta n \quad (2)$$

where d is the sample thickness.

When two different birefringent materials 1 and 2 are stacked, the total retardation is equal to the sum of their retardations, i.e.,

$$R = R_1 + R_2 \quad (3)$$

Interference colors can be observed for birefringent materials under crossed polarizers because of retardation. The HAp liquid crystal was observed by POM with a wave plate with a retardation of 530 nm (Supplementary Fig. 8). When the orientation direction of the liquid crystal is identical to the Z' direction of the wave plate, blue interference is observed (Supplementary Fig. 8a). This blue color indicates that the retardation of the liquid crystal was added to that of the wave plate. In contrast, when the orientation direction becomes perpendicular to the Z' direction of the wave plate, the interference color changes to yellow (Supplementary Fig. 8c). This means that the retardation of the liquid crystal was subtracted from that of the wave plate. These interference color changes indicate positive birefringence of the LC HAp material ($n_{||} > n_{\perp}$)[4].

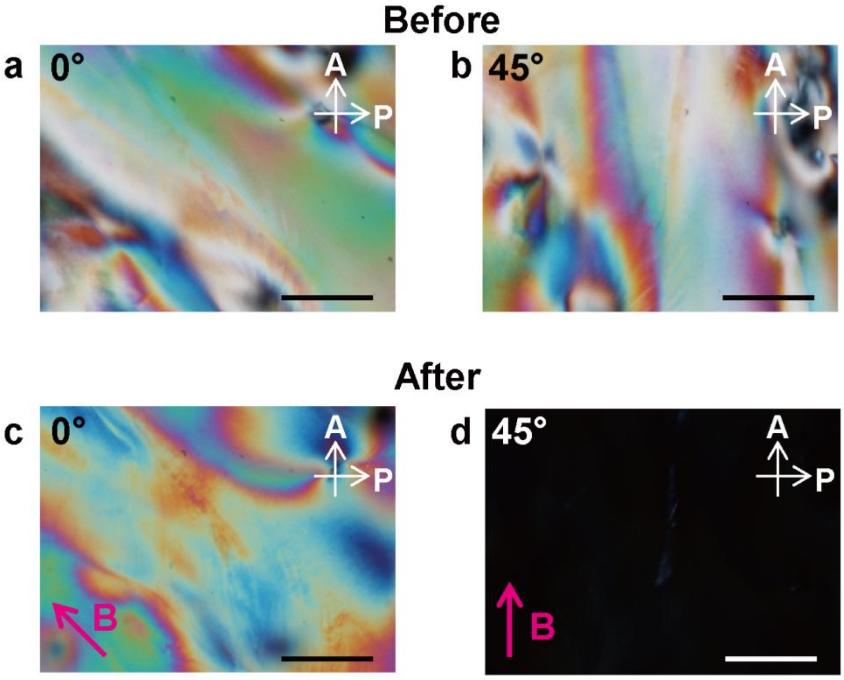

Supplementary Figure 9 POM images of LC aqueous colloidal dispersion before and after application of 3 T magnetic field. **a–d** POM images for a LC aqueous colloidal dispersion (9.4 vol%) (**a, b**) before and (**c, d**) after application of a 3 T magnetic field. Parts (b) and (d) are POM images of samples rotated clockwise by 45° from the positions in (**a**) and (**c**), respectively. The magenta arrows in (**c**) and (**d**) indicate the direction of the applied magnetic field. A: Analyzer; P: Polarizer. Scale bars, 500 μm.

Supplementary Note 9

The LC colloidal dispersion of HAp nanorods showed randomly oriented domains before the application of a magnetic field (Supplementary Fig. 9a, b). After application of a 3 T magnetic field, repeated bright and dark images were observed under crossed polarizers on each rotation of the sample by 45° (Supplementary Fig. 9c, d), indicating that unidirectional macroscopic alignment was generated by the magnetic field.

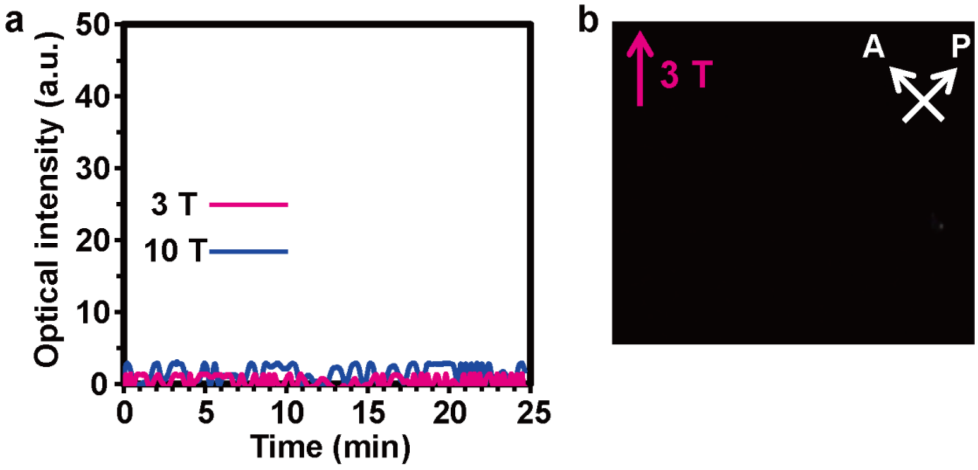

Supplementary Figure 10 Dynamics of magnetic responses of isotropic (Iso) aqueous colloidal dispersion at 3 T and 10 T. **a** Time courses of responses of a Iso aqueous colloidal dispersion (4.2 vol%) at 3 T and 10 T. **b** *In situ* observation under crossed polarizers in the presence of a 3 T magnetic field. The magenta arrow indicates the direction of the magnetic field. A: Analyzer; P: Polarizer.

Supplementary Note 10

The Iso colloidal dispersion of HAp nanorods did not respond to a 3 T magnetic field or a magnetic field of 10 T (Supplementary Fig. 10a). The dark image under crossed polarizers indicates random orientation of the HAp nanorods (Supplementary Fig. 10b).

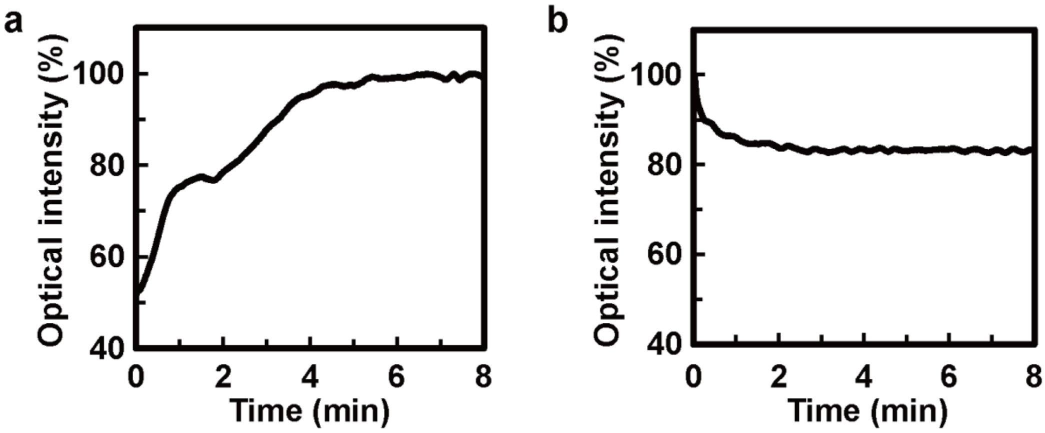

Supplementary Figure 11 Dynamics of magnetic response and relaxation of LC colloidal dispersions in 2-mm-thick optical cell. **a** Time course of response of a LC aqueous colloidal dispersion (9.4 vol%) at 3 T and **b** relaxation behavior after the magnetic field of 3 T was turned off.

Supplementary Note 11

A LC colloidal dispersion of HAp nanorods in a 2-mm-thick optical cell took around 5 min to reach maximum light transmission (Supplementary Fig. 11a). The relaxation process was complete in 3 min after removal of the magnetic field, although the optical intensity did not decrease to the original value before application of the magnetic field (Supplementary Fig. 11b).

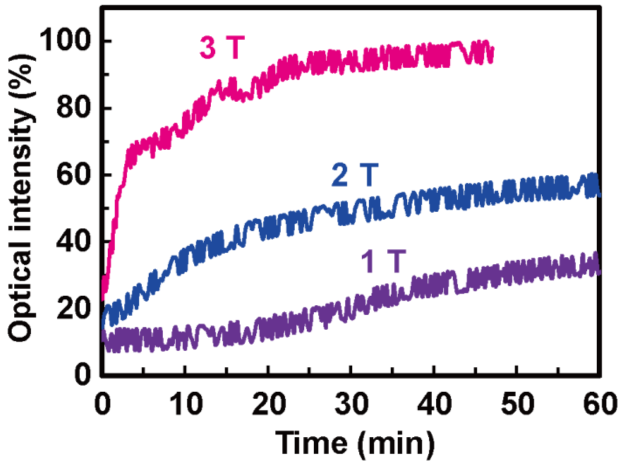

Supplementary Figure 12 Dynamics of magnetic responses of LC aqueous colloidal dispersion in 0.1-mm-thick cell. Time courses of responses of a LC aqueous colloidal dispersion (9.4 vol%) under 1 T, 2 T, and 3 T magnetic fields.

Supplementary Note 12

A LC colloidal dispersion of HAp nanorods in a 0.1-mm-thick cell took around 40 min to reach the maximum light transmission at 3 T. Under 2 T and 1 T magnetic fields, a longer time, i.e., several hours, was required to reach equilibrium (Supplementary Fig. 12).

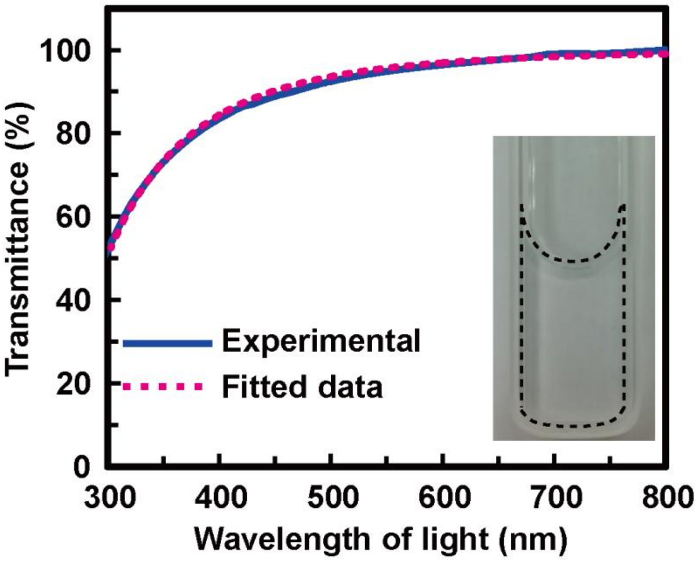

Supplementary Figure 13 Optical properties of Iso/LC biphasic aqueous colloidal dispersion. The transmittance spectrum of a biphasic aqueous colloidal dispersion (8.5 vol%) in a 2-mm-thick optical cell was recorded as a function of light wavelength (blue solid line). The magenta dashed line is the fitted data calculated based on the Rayleigh scattering equation. The inset shows a digital photograph of the colloidal dispersion in a 2-mm-thick optical cell.

Supplementary Note 13

The Iso/LC biphasic colloidal dispersion showed high optical transparency in the visible light wavelength range (Supplementary Fig. 13, blue solid line). The optical transmittance was 98% at 670 nm, even in a 2-mm-thick cell, indicating that the light loss was extremely small. The experimental data for the transmittance was reasonably fitted by the following Supplementary Equation (4), which represents Rayleigh scattering (Supplementary Fig. 13, magenta dashed line):

$$T = 1 - \left(\frac{k_{\mathrm{s}}}{\lambda^4}\right) \tag{4}$$

where k_{s} is the scattering coefficient and λ is the light wavelength.

These results show that no unfavorable light absorption and scattering were generated in this dispersion.

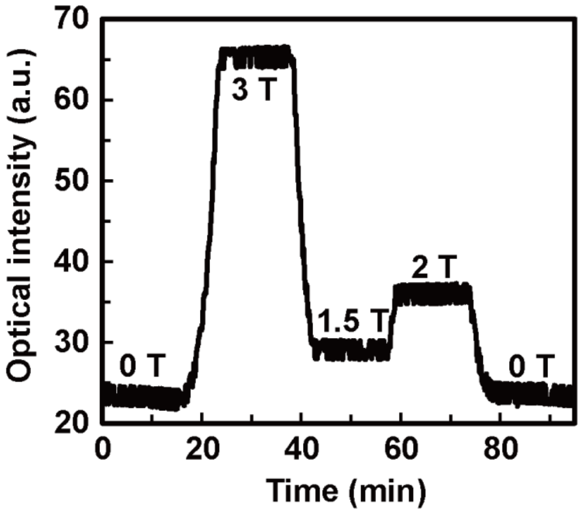

Supplementary Figure 14 Modulation of transmitted light intensity at low field strength. Change in the intensity of light transmitted through a 8.5 vol% Iso/LC biphasic aqueous colloidal dispersion between crossed polarizers by tuning the field strength below 3 T when a light source with increased intensity was used. The rate of change of the magnetic field strength was 0.36 T min^{-1}.

Supplementary Note 14

Light transmission was successfully modulated for the Iso/LC biphasic colloidal dispersion at lower field strengths, i.e., between 1 and 3 T, using a light source with increased intensity (Supplementary Fig. 14).

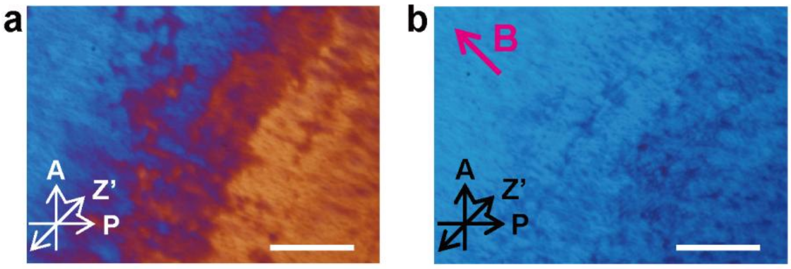

Supplementary Figure 15 Interference color observations for LC aqueous colloidal dispersion aligned by magnetic field. **a, b** POM images of a LC aqueous colloidal dispersion (9.4 vol%) in a 0.1-mm-thick cell observed in presence of a wave plate (**a**) before and (**b**) after application of a 3 T magnetic field. The magenta arrow in (**b**) indicates the direction of the applied magnetic field. A: Analyzer; P: Polarizer; Z’: slow axis of the wave plate. Scale bars, 500 μm.

Supplementary Note 15

The interference color of a LC colloidal dispersion with a sample thickness of 0.1 mm (Supplementary Fig. 15a) changed to uniform blue when the applied magnetic field was perpendicular to the *Z*' direction of the wave plate (Supplementary Fig. 15b). Based on the positive birefringence of the HAp liquid crystal (Supplementary Fig. 8), the alignment direction of the HAp nanorods was perpendicular to the magnetic field direction. This orientation behavior is consistent with the fact that the *c* axis has a smaller magnetic susceptibility in the HAp crystal[5].

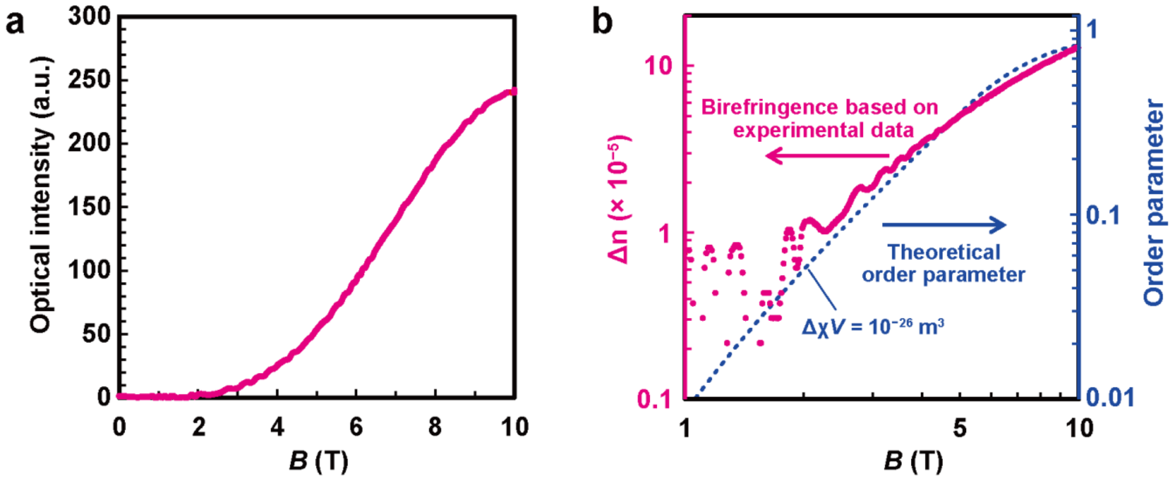

Supplementary Figure 16 Estimation of anisotropy of magnetic susceptibility of LC droplet in Iso/LC biphasic colloidal dispersion. **a** Change in the intensity of light transmitted through a Iso/LC biphasic colloidal dispersion (8.5 vol%) under crossed polarizers as a function of applied magnetic fields. **b** Fitting of birefringence calculated from experimental data in (**a**) (magenta dots), and the theoretical curve for the order parameter as a function of applied magnetic fields obtained by setting $\Delta\chi V$ as the fitting parameter (blue dashed line).

Supplementary Note 16

Under the present experimental condition, the birefringence is derived from the transmitted light intensity according to the following Supplementary Equations (5,6):

$$I = I_{\mathrm{max}}\sin^2\frac{\pi d\Delta n}{\lambda} \tag{5}$$

$$\Delta n = \frac{\lambda}{\pi d}\sin^{-1}\sqrt{\frac{I}{I_{\mathrm{max}}}} \tag{6}$$

Here, d is the sample thickness, Δn is the birefringence, and λ is the wavelength of the light. The birefringence can also be described as a function of the order parameter S:

$$\Delta n = \Delta n_{\mathrm{inherent}}S \tag{7}$$

where $\Delta n_{\mathrm{inherent}}$ is the birefringence when the material is perfectly oriented.

The order parameter of a particle under a magnetic field is theoretically calculated by setting $\Delta\chi V$ values as the fitting parameter based on the following Supplementary Equations (8–10) representing the anisotropic magnetic energy, the orientational distribution function and the orientational order parameter, respectively[6].

$$E_{\mathrm{m_{ani}}}(\theta) = -\frac{V\chi_{\perp}B^2}{2\mu_0} - \frac{V\Delta\chi(B\cos\theta)^2}{2\mu_0} \tag{8}$$

$$P(\theta)\sin\theta\cdot d\theta = \frac{\exp(-E(\theta)/k_{\mathrm{B}}T)\sin\theta\cdot d\theta}{\int_0^{\pi/2}\exp\,(-E(\theta)/k_{\mathrm{B}}T)\sin\theta\cdot d\theta} \tag{9}$$

$$S = \frac{3\langle\cos^2(\theta)\rangle - 1}{2} \tag{10}$$

Here, V is the volume of the particle, $\chi_{\perp}$ is the magnetic susceptibility in the direction perpendicular to the long axis of the particle, μ_0 is the magnetic permeability of a vacuum, and θ is the angle between the magnetic field and the long axis of the particle.

The birefringence of the Iso/LC colloidal dispersion (Supplementary Fig. 16b, magenta dots) was derived from the experimental results for the transmitted light intensity (Supplementary Fig. 16a), based on Supplementary Equation (6), by approximating the maximum optical intensity, I_{max}, to the value at 10 T. The birefringence is a function of the order parameter as expressed by Supplementary Equation (7), therefore the birefringence data based on the optical intensity measurements were fitted to the theoretical data for the order parameter calculated using Supplementary Equation (10) by choosing 10^{-26} m^3 as the value of $\Delta\chi V$ (Supplementary Fig. 16b). If we assume that the average size of a LC droplet in the biphasic colloidal dispersion is 1 $\mu\mathrm{m}^3$ (10^{-18} m^3), the anisotropy of the magnetic susceptibility $\Delta\chi$ is roughly estimated to be -10^{-8}.

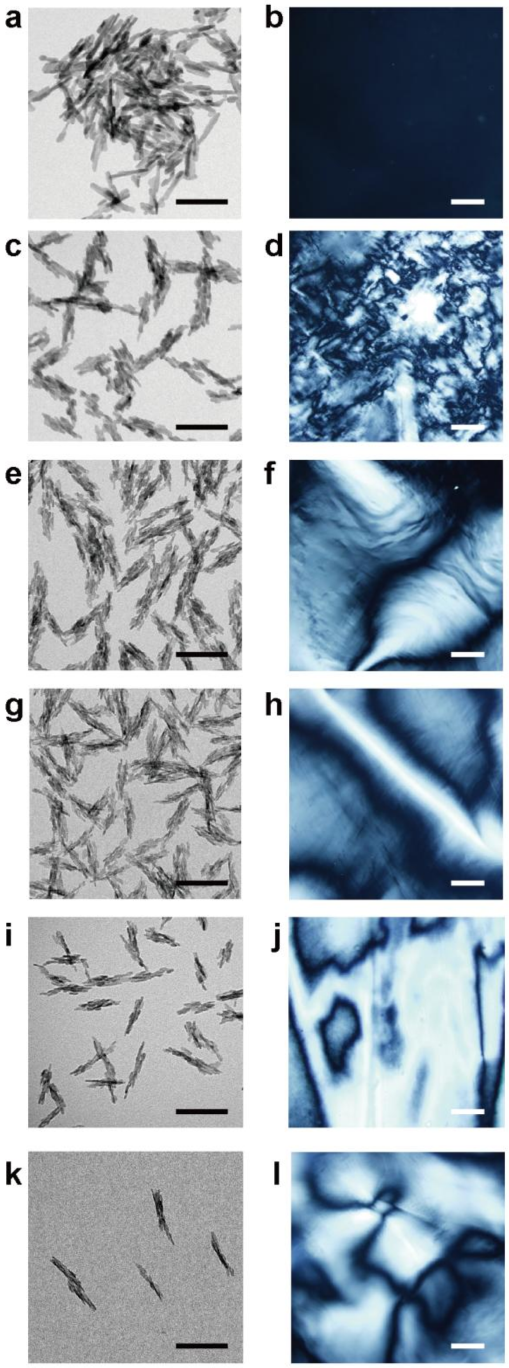

Supplementary Figure 17 TEM images of crystals synthesized at various PAA concentrations and POM images of the condensed aqueous dispersions. **a, c, e, g, i, k** TEM images of crystals and **b, d, f, h, j, l** POM images of condensed aqueous dispersions of the crystals synthesized at different PAA concentrations of (**a, b**) 0 wt%, (**c, d**) 0.18 wt%, (**e, f**) 0.36 wt%, (**g, h**) 0.54 wt%, (**i, j**) 0.72 wt% and (**k, l**) 0.90 wt%. The concentrations of HAp crystals in these aqueous dispersions are (**b**) 13 vol%, (**d**) 15 vol%, (**f**) 18 vol%, (**h**) 11 vol%, (**j**) 9.2 vol% and (**l**) 13 vol%, respectively. Scale bars, (**a, c, e, g, i, k**) 100 nm and (**b, d, f, h, j, l**) 200 μm.

Supplementary Note 17

The TEM and POM observations show that LC HAp nanorods were synthesized in the PAA concentration range 0.18–0.90 wt% (Supplementary Fig. 17). The average length and width of these LC nanorods were 120 ± 30 nm and 32 ± 8 nm (Supplementary Fig. 17c), 120 ± 30 nm and 31 ± 7 nm (Supplementary Fig. 17e), 100 ± 20 nm and 27 ± 6 nm (Supplementary Fig. 17g), 100 ± 20 nm and 21 ± 5 nm (Supplementary Fig. 17i) and 90 ± 20 nm and 23 ± 5 nm (Supplementary Fig. 17k), respectively. The size and aspect ratios of these LC nanorods were similar. The viscosities of LC colloidal dispersions of nanorods showed an increasing trend as the concentrations of PAA decrease.

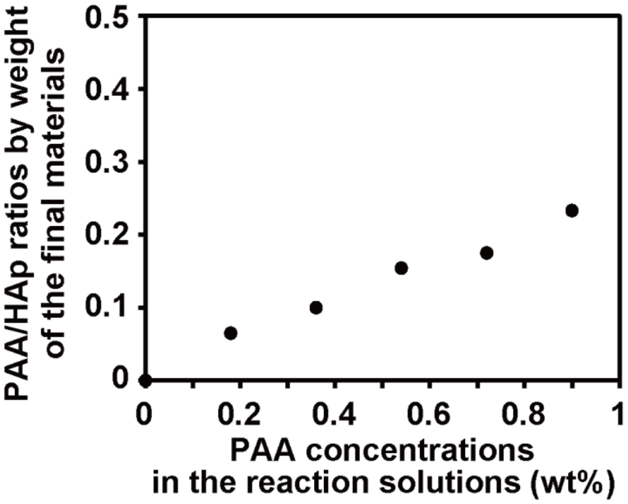

Supplementary Figure 18 Composition analyses of HAp crystals synthesized at various PAA concentrations. PAA/HAp ratios by weight were estimated for final materials synthesized at various PAA concentrations based on TG measurements.

Supplementary Note 18

The amounts of PAA molecules self-assembled with HAp crystals were decreased with decreasing PAA concentrations in the reaction solutions (Supplementary Fig. 18).

Supplementary references

1. Jiang, S., Pan, H., Chen, Y., Xu, X. & Tang, R. Amorphous calcium phosphate phase-mediated crystal nucleation kinetics and pathway. *Faraday Discuss.* **179**, 451–461 (2015).

2. Mcneill, I. C. & Sadeghi, S. M. T. Thermal stability and degradation mechanisms of poly (acrylic acid) and its salts : part 1 poly (acrylic acid). *Poly. Deg. and Stab.* **29**, 233–246 (1990).

3. Tagaya, A., Ohkita, H., Mukoh, M., Sakaguchi, R. & Koike, Y. Compensation of the birefringence of a polymer by a birefringent crystal. *Science* **301**, 812–814 (2003).

4. Murata, K. & Haraguchi, K. Optical anisotropy in polymer–clay nanocomposite hydrogel and its change on uniaxial deformation. *J. Mater. Chem.* **17**, 3385–3388 (2007).

5. Akiyama, J. *et al.* Formation of c-axis aligned polycrystal hydroxyapatite using a high magnetic field with mechanical sample rotation. *Mater. Trans.* **46**, 203–206 (2005).

6. Asai, S. *et al.* in *Magneto-Science, Magnetic Field Effects on Materials: Fundamentals and Applications.* Ch. 5 (eds Yamaguchi, M. & Tanimoto Y.) (KODANSHA/Springer, 2006).
